# Factors influencing COVID-19 vaccine uptake among adults in Nigeria

**DOI:** 10.1371/journal.pone.0264371

**Published:** 2022-02-24

**Authors:** Halimat Adedeji-Adenola, Olubusola A. Olugbake, Shakirat A. Adeosun

**Affiliations:** 1 Discipline of Pharmaceutical Sciences, University of KwaZulu- Natal, Durban, South Africa; 2 Directorate of Pharmaceutical Services, Lagos State Primary Healthcare Board, Lagos, Lagos State, Nigeria; 3 Faculty of Pharmacy, University of Lagos, Lagos, Lagos State, Nigeria; University of South Carolina College of Pharmacy, UNITED STATES

## Abstract

**Background:**

Emerging variants of Coronavirus disease 2019 (COVID-19) has claimed over 3000 lives in Nigeria and vaccination remains a means of reducing the death toll. Despite ongoing efforts by the government to ensure COVID-19 vaccination of most residents to attain herd immunity, myths and beliefs have adversely shaped the perception of most Nigerians, challenging the uptake of COVID-19 vaccine. This study aimed to assess the factors influencing the awareness, perception, and willingness to receive COVID-19 vaccine among Nigerian adults.

**Methods:**

A cross-sectional online nationwide study was conducted from April to June 2021 among Nigerian adult population using the snowballing method. Descriptive analysis was used to summarise the data. Univariate and multivariate analysis was used to identify the predictors of COVID-19 uptake among the respondents. A p value <0.05 was considered significant.

**Results:**

A total of 1058 completed forms were analysed and 63.9% were females. The mean age was 40.8 years±12.2 years. Most of the respondents (740; 69.5%) had satisfactory awareness of the vaccination exercise. The media was the main source of information. Health workers reported higher level of awareness (aOR = 1.822, 95% CI: 1.388–2.524, p<0.001). Respondents that are Christians and Muslims had better awareness compared to the unaffiliated (aOR = 6.398, 95% CI: 1.918–21.338, P = 0.003) and (aOR = 7.595, 95% CI: 2.280–25.301, p<0.001) respectively. There is average score for perception statements (566; 53.2%) towards COVID-19 vaccination. Close to half of the respondents (44.2%) found the short period of COVID-19 production worrisome. Majority of the respondents were willing to get the vaccine (856; 80.9%). Those without a prior diagnosis of COVID-19 had a lower willingness to get vaccinated (aOR = 0.210 (95% CI: 0.082–0.536) P = 0.001).

**Conclusion:**

The study revealed a high level of awareness, willingness to receive the vaccine and moderate perception towards the vaccination activities. Influencing factors that significantly affects awareness were religion, occupation, education and prior diagnosis of COVID-19; for perception and willingness—occupation, and prior diagnosis of the COVID-19 were influencing factors.

## Introduction

Coronavirus disease 2019 (COVID-19) has been described as a global health pandemic that has the potential to cause unprecedented devastating socio, economic and political crises [1]. To stem the ongoing COVID-19 pandemic, Nigeria received an estimated 4 million doses of the AstraZeneca/Oxford vaccine through the COVID-19 Vaccines Global Access (COVAX) facility, a partnership between Coalition for Epidemic Preparedness Innovations (CEPI), Global Alliance for Vaccines and Immunizations (GAVI), United Nation Children’s Fund (UNICEF) and World Health Organisation (WHO) in March 2021 [1]. The National Primary Health Care Development Agency (NPHCDA), through the State’s Primary Health Care Board, commenced the vaccination of Nigerians’ priority groups in March 2021, starting with frontline healthcare workers, strategic leaders, security officials and other public personnel identified as eligible for the first phase of vaccination [2]. While the government is doing its best to ensure that most Nigerians gets vaccinated, there has been hope, fear, government distrust, hesitancy, rejection, and conspiracy theories as the COVID-19 vaccination exercise continues [3]. Studies both globally and locally have shown that there is a diverse rate of variability in the awareness, perception, willingness, and acceptance rate of the COVID -19 vaccine [4–6]. In the United States, willingness to vaccinate declined during the pandemic compared with Malaysia and Bangladesh who generally had more positive attitudes to receiving the COVID-19 vaccine [5, 7, 8]. In a study carried out in Australia to understand the perception towards future COVID-19 vaccination, the majority of the public held positive view [9]. A study in Bangladesh showed vaccine uptake was affected by inadequate knowledge and perception of respondents [10]. Nigeria and Ghana had just over 50% of the population studied as willing to take the vaccine and recommend it to others [11–14]. In the United Kingdom, Black African and Black Caribbean groups were less likely to be vaccinated (50%) compared to White groups (70%) [14, 15]. Another study in Yemen found that vaccine acceptance was significantly affected by gender, misinformation, cost, and income [16]. In a survey carried out before the commencement of the vaccination exercise in Nigeria, respondents showed poor perception and a lots were unwilling to participate in COVID-19 vaccine trial [6].

Generally, for a successful immunization program, there is a need to have a high rate of vaccination coverage. It is important to understand Nigerian factors that influence awareness, perception, and willingness to receive the vaccine especially as the first phase of the vaccination exercise kicked off with health workers and frontline personnel. It is hoped that this will guide towards planning for the subsequent phases that will involve a larger population of Nigerians.

The aim of this study was to assess the factors influencing the awareness, perception, and willingness to receive the COVID-19 vaccination among adults in Nigeria.

## Methods

### Study design

A cross-sectional study was conducted from April to June 2021. A structured self-administered questionnaire was designed and incorporated into the Google survey tool (Google Forms). This was shared with the public on social media such as Facebook, WhatsApp, Telegram, Twitter, and LinkedIn using the generated link.

### Study participants, sampling size and sampling

The study targeted eligible Nigerian residents. A sample size of 664 participants was computed for a population of 212million Nigerians [17] at a 99% confidence level, 5% margin of error and 50% response rate using the Open Source Epidemiologic Statistics for Public Health (OpenEpi), version 3 [18]. Participants were recruited using the Snowball sampling technique. The researchers posted the questionnaire on the various social media groups and in turn, these prospective respondents were encouraged to send to their own contacts and online platforms. Respondents were recruited irrespective of gender, cultural background, or origin, aged 18years and above while respondents that completed the questionnaire outside Nigeria were excluded. Those that did not consent could not complete the electronic questionnaire as it was a requirement to attempt the questions.

### Data collection and analysis

The data collection tool was adapted from similar studies [4, 9, 19] and frequently asked questions in the media about COVID-19 vaccination. The questionnaire prepared in the English language took an average of 5 minutes to be completed. Participants were assured of confidentiality. The e- survey collected data on the following: socio-demographic information such as gender, age, religion, highest education level attained, residence, history of a chronic condition, history of travel, monthly income, and occupation (categorised as health workers and non- health workers); Awareness of participants regarding the first phase of COVID-19 vaccination had 4 items with a Yes or No options, willingness to receive the vaccine had 1 item. Also, information on the perceptions of respondents to the COVID-19 vaccine had10 items on a 5- point Likert scale (Strongly agree, agree, not sure, disagree and strongly disagree). The questionnaire was assessed for content validity by clinical pharmacists. A pilot study was carried out among 10 randomly selected social media participants for clarity, acceptability, and readability. This led to changes and modifications of some items and options before the final survey was distributed to the study population.

The data were sorted, coded, and analysed using IBM SPSS statistics version 24.0 software and Microsoft Excel 365. To summarize the data, descriptive statistics such as frequency and percentage were used for the sociodemographic characteristics. A numeric scoring pattern was used to assess the awareness, willingness, and perception levels of respondents to the first phase of the vaccination exercise. The outcome variables (awareness, perception, and willingness) were further categorized as ‘satisfactory’ or ‘unsatisfactory’ based on their individual mean scores from a maximum obtainable score which serve as cut-off marks. Scores above the mean scores obtained for these outcome variables were classified as ‘satisfactory responses’ and vice versa [20]. Inferential statistics (logistic regression model) was used. The logistic regression model was used to identify factors that influence awareness, perception, and willingness to get the COVID-19 vaccine. Univariate associations were ascertained with each demographic variable and the outcome variable (Crude odds ratio; cOR). A multivariable logistic regression model was also created to estimate associations (adjusted odds ratios; aOR) and 95% confidence intervals (CIs) with significance set at p = 0.05 were used [21].

### Ethical consideration

Ethics approval was granted by the Health Research and Ethics Committee of Lagos University Teaching Hospital, Nigeria (HREC, LUTH) with approval number: ADM/DSCT/HREC/APP/4234. Participants in the study were informed about the procedure and purpose of the study in the first section on the Google form. Confidentiality of information provided was assured. All participants consented willingly to be a part of the study during the data collection periods by clicking on the consent button. All the data was collected anonymously and analyzed using the coding system.

## Results

A total of 1063 forms were retrieved, however,1058 completed forms were analysed in this study. There were more female respondents (676; 63.9%) than males (382; 36.1%). The mean age was 40.8 years ± 12.2 years. Most respondents have bachelor/master’s degrees (801; 75.7%). There were more health workers (594; 56.1%) than non-health workers (464; 43.9%) (Table 1).

**Table 1:** Sociodemographic characteristics of respondents (N = 1058).

Variables		Frequency	N (%)
Gender	Female	676	63.9
	Male	382	36.1
Age- range	18–29	193	18.2
	30–39	325	30.7
	40–49	265	25.0
	50–59	208	19.7
	60–69	50	4.7
	>70	17	1.6
Highest level of education	No formal education	5	0.5
	Primary	4	0.4
	Secondary	54	5.1
	Diploma	194	18.3
	Bachelor’s degree	429	40.5
	Masters and above	372	35.2
Religion	Christianity	543	51.3
	Islam	499	47.2
	Traditional	1	0.1
	Unaffiliated	15	1.4
Occupation	Health workers	594	56.1
	Non-health workers	464	43.9
Chronic condition	No	970	91.7
	Yes	88	8.3
Travel in last 12 months	No	974	92.1
	No, but I will travel soon	45	4.3
	Yes	39	3.7
Prior diagnosis of COVID-19	No	952	90.0
	Yes	106	10.0

### Awareness, perception, and willingness towards COVID-19 vaccine

**Fig 1:**
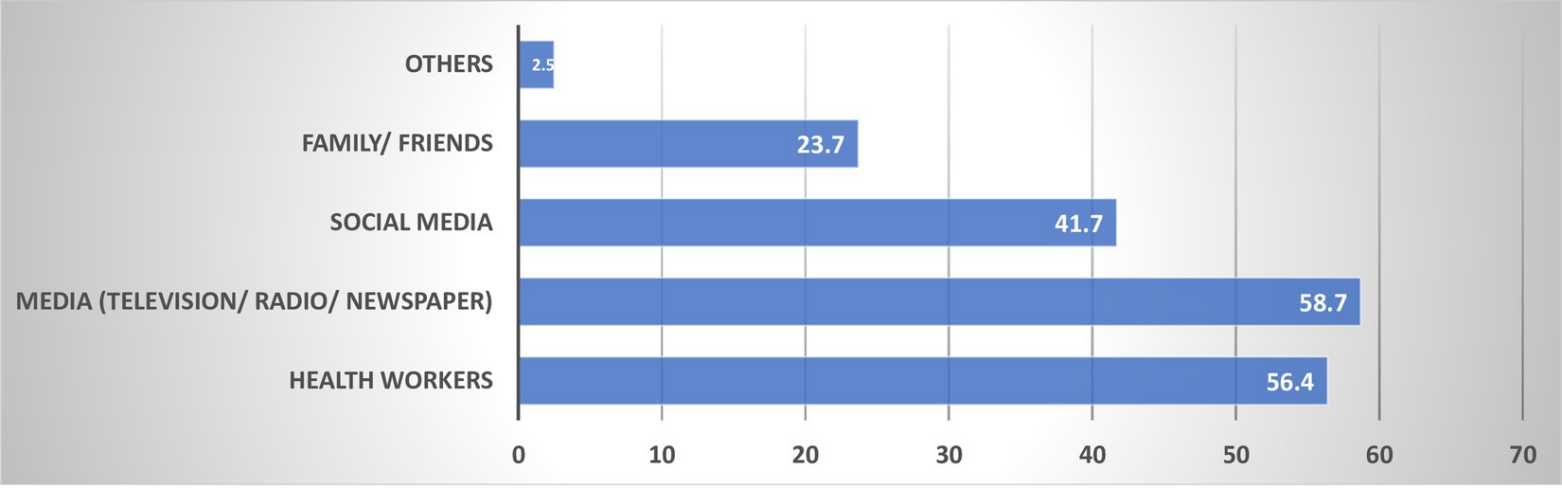
Respondents’ sources of information of first phase COVID-19 vaccination exercise in Nigeria. Individual responses may have multiple sources of information. The total sources of COVID-19 vaccination information are not equal to the total or respondents.

The mean awareness score was 2.9±0.8 (Table 2). Most of the respondents (740; 69.5%) had satisfactory awareness of the first phase vaccination exercise. The media was the main source of information (Fig 1).

**Table 2:** Description of outcome variable scores obtained by respondents (n = 1058).

Outcome variables	Maximum obtainable scores	Scores received by respondents		Mean ± SD	Satisfactory n (%)	Unsatisfactory n (%)
		Minimum score	Maximum score			
Awareness	4	0	4	2.9 ±0.8	740 (69.5%)	318 (29.9%)
Perception	50	14	50	35.0 ±6.5	566 (53.2%)	492 (49.2%)

Cut-off marks = mean scores i.e., awareness- 2.9, perception- 35.0. Satisfactory scores = scores > mean score obtained by respondents. SD- standard deviation.

The perception of participants (566; 53.2%) towards COVID-19 vaccination was satisfactory with a mean perception score of 35.0 ±6.5 (Table 2). Most respondents accepted that the COVID-19 vaccine is effective at preventing the disease (814; 76.9%, obtained as sum of strongly agree and agree). Some respondents consented that the short period of COVID-19 production is worrisome (468; 44.2%, obtained as sum of strongly agree and agree) while some are neutral to this statement (311; 29.4%). On the perception that vaccines produced for Europe and America are safer, respondents perceived (465; 44%, obtained as sum of strongly agree and agree) while some respondents were neutral to this statement (390; 36.9%). Most of the respondents were willing to get the COVID-19 vaccine (856; 80.9%).

### Associated sociodemographic factors influencing the outcome variables (awareness, perception, and willingness) of COVID-19 vaccination

Sociodemographic factors such as occupation, prior diagnosis of COVID-19, education and religion were statistically significant on awareness of respondents of the first phase of COVID-19 vaccination exercise. Health workers reported higher level of awareness (aOR = 1.87 (95% CI: 1.13–2.53) P< 0.001) than non- health workers. Those without prior diagnosis of COVID-19 have lesser awareness (aOR = 0.56 (95% CI: 0.33–0.95) P = 0.031) than those that have been tested positive to COVID-19 disease. Participants with diploma education had higher level of awareness (aOR = 2.24 (95% CI: 1.05–4.59) P = 0.028). Respondents who are Christians and Muslims had better awareness than others (aOR = 6.36 (95% CI: 1.91–21.20) P = 0.003) and (aOR = 7.55 (95% CI: 2.27–25.16) P = 0.001) respectively (Table 3).

**Table 3:** Sociodemographic characteristics influencing the level of awareness of respondents towards the COVID-19 vaccination.

Variable		Satisfactory (%)	Unsatisfactory (%)	cOR (95%CI)	P value	aOR (95%CI)	P value
Gender	Female	484 (45.7)	192 (18.1)	1.24(0.59–1.63)	0.119	1.08(0.81–1.45)	0.604
	Male	256 (24.2)	126 (11.9)	1	Ref	1	Ref
Age range	18–29	123 (11.6)	70 (6.6)	0.97(0.34–2.73)	0.948	0.98(0.33–2.87)	0.966
	30–39	230 (21.7)	95 (9.0)	1.30(0.47–3.63)	0.612	1.21(0.42–3.49)	0.727
	40–49	184 (17.4)	81 (7.7)	1.25(0.45–3.48)	0.675	1.26(0.44–3.66)	0.666
	50–59	155 (14.7)	53 (5.0)	1.61(0.57–4.55)	0.373	1.45(0.50–4.25)	0.500
	60–69	37 (3.5)	13 (1.2)	1.55(0.48–5.05)	0.464	2.08(0.60–7.21)	0.250
	>70	11 (1.0)	6 (0.6)	1	Ref	1	Ref
Religion	Christianity	387 (36.6)	156 (14.7)	6.82(2.14–21.75)	**0.001** *	6.36(1.91–21.20)	**0.003** *
	Traditional	1 (0.1)	0 (0.0)	λ	1.000	λ	1.000
	Islam	348 (32.9)	151 (14.3)	6.34(1.99–20.22)	**0.002** *	7.55(2.27–25.16)	**0.001** *
	Unaffiliated	4 (0.4)	11 (1.0)	1	Ref	1	Ref
Education	No formal education	0 (0.0)	5 (0.5)	0.00(0.00)	0.999	0.00(0.00)	0.999
	Primary	3 (0.3)	1 (0.1)	1.80(1.01–3.20)	0.461	1.16(0.62–2.16)	0.661
	Secondary	30 (2.8)	24 (2.3)	1	Ref	1	Ref
	Diploma	161 (15.2)	33 (3.1)	3.90(2.03–7.51)	**0.000** *	2.24(1.09–4.59)	**0.028** *
	Bachelor’s degree	297 (28.1)	132 912.5)	1.62(0.91–2.89)	**0.045** *	1.01(0.52–1.95)	0.652
	Masters and above	249 (23.5)	123 (11.6)	2.40(0.23–24.57)	0.102	1.72(1.54–19.06)	0.984
Occupation	Health worker	459 (43.4)	136 (12.9)	2.19(1.67–2.86)	**0.000** *	1.87(1.39–2.53)	**0.000** *
	Non-health worker	281 (26.6)	182 (17.2)	1	Ref	1	Ref
Chronic condition	No	671 (63.4)	299 (28.3)	0.62(0.37–1.05)	0.073	0.65(0.38–1.13)	0.128
	Yes	69 (6.5)	19 (1.8)	1	Ref	1	Ref
Prior diagnosis of COVID-19	No	654 (61.8)	298 (28.2)	0.51(0.31–0.85)	**0.009** *	0.56(0.33–0.95)	**0.031** *
	Yes	86 (8.1)	20 (1.9)	1	Ref	1	Ref

cOR = crude odds ratio, aOR = adjusted odds ratio, CI = confidence interval, λ = not included in comparison due to small number

* = statistically significant.

On perception towards COVID-19 vaccination, respondents that are health workers had better perception (aOR = 2.86 (95% CI: 2.15–3.79) P< 0.001) than non- health workers. Those without prior diagnosis of COVID-19 had lesser perception (aOR = 0.59 (95% CI: 0.38–0.92) P = 0.021) than those that were previously COVID-19 positive (Table 4).

**Table 4:** Associated sociodemographic factors influencing the perception of COVID-19 vaccination.

Variable		Satisfactory (%)	Unsatisfactory (%)	cOR (95%CI)	P value	aOR (95%CI)	P value
Gender	Female	362 (34.2)	314 (29.7)	1.00(0.78–1.29)	0.963	0.82(0.62–1.08)	0.157
	Male	204 (19.3)	178 (16.8)	1	Ref	1	Ref
Age range	18–29	71 (6.7)	122 (11.5)	0.68(0.25–1.84)	0.446	0.66(0.23–1.89)	0.442
	30–39	166 (15.7)	159 (15.0)	1.14(0.43–3.03)	0.794	0.90(0.32–2.51)	0.842
	40–49	146 (13.8)	119 (11.2)	1.39(0.52–3.71)	0.512	1.19(0.43–3.33)	0.742
	50–59	142 (13.4)	66 (6.2)	2.44(0.90–6.60)	0.080	1.83(0.65–5.19)	0.256
	60–69	33 (3.1)	17 (1.6)	2.18(0.71–6.68)	0.171	2.53(0.78–8.25)	0.123
	>70	8 (0.8)	9 (0.9)	1	Ref	1	Ref
Religion	Christianity	323 (30.5)	220 (20.8)	2.20(0.77–6.28)	0.139	1.97(0.65–5.99)	0.230
	Traditional	0 (0.0)	1 (0.1)	**λ**	1.000	**λ**	1.000
	Islam	237 (22.4)	262 (24.8)	1.36(0.48–3.87)	0.568	1.63(0.54–4.93)	0.387
	Unaffiliated	6 (0.6)	9 (0.9)	1	Ref	1	Ref
Education	No formal education	0 (0.0)	5 (0.5)	**λ**	0.999	**λ**	0.999
	Primary	2 (0.2)	2 (0.2)	2.52(1.37–4.61)	0.455	1.04(0.53–2.03)	0.786
	Secondary	17 (1.6)	37 (3.5)	1	Ref	1	Ref
	Diploma	113 (10.7)	81 (7.7)	3.04(1.60–5.77)	**0.001** *	1.07(0.52–2.18)	0.862
	Bachelor’s degree	230 (21.7)	199 (18.8)	2.64(1.44–4.86)	**0.003** *	1.00(0.50–1.99)	0.907
	Masters and above	204 (19.3)	168 (15.9)	2.18(0.28–16.78)	**0.002** *	0.74(0.09–6.43)	0.990
Occupation	Health worker	392 (37.1)	203 (19.2)	3.21(2.49–4.13)	**0.000** *	2.86(2.15–3.79)	**0.000** *
	Non-health worker	174 (16.4)	289 (27.3)	1	Ref	1	Ref
Chronic condition	No	515 (48.7)	455 (43.0)	0.82(0.53–1.28)	0.382	1.03(0.63–1.68)	0.897
	Yes	51 (4.8)	37 (3.5)	1	Ref	1	Ref
Prior diagnosis of COVID-19	No	495 (46.8)	457 (43.2)	0.53(0.35–0.82)	**0.004** *	0.59(0.38–0.92)	**0.020** *
	Yes	71 (6.7)	35 (3.3)	1	Ref	1	Ref

cOR = crude odds ratio, aOR = adjusted odds ratio, CI = confidence interval, λ = not included in comparison due to small number

* = statistically significant.

On willingness to receive COVID-19 vaccine, agreement was higher for those who are health workers in this survey (aOR = 4.83 (95% CI: 3.28–7.11) P< 0.001) than non-health workers. Also, there was low willingness to receive the vaccine among those without prior COVID-19 diagnosis (aOR = 0.21 (95% CI: 0.08–0.54) P = 0.001) as compared to those that previously had the virus (Table 5).

**Table 5:** Associated sociodemographic factors influencing the willingness to receive the COVID-19 vaccine.

Variable		Yes (%)	No (%)	cOR (95%CI)	P value	aOR (95%CI)	P value
Gender	Female	548 (51.8)	128 (12.1)	1.01(0.73–1.39)	0.944	0.74(0.52–1.07)	0.107
	Male	309 (29.2)	73 (6.9)	1	Ref	1	Ref
Age range	18–29	148 (14.0)	45 (4.3)	0.99(0.31–3.19)	0.987	0.81(0.22–2.96)	0.748
	30–39	262 (24.6)	63 (6.0)	1.29(0.41–4.09)	0.665	0.78(0.22–2.80)	0.704
	40–49	210 (19.8)	55 (5.2)	1.18(0.37–3.76)	0.779	0.86(0.24–3.07)	0.814
	50–59	181 (17.1)	27 (2.6)	2.07(0.63–6.83)	0.230	1.34(0.36–4.97)	0.659
	60–69	43 (4.1)	7 (0.7)	1.89(0.48–7.89)	0.365	2.48(0.52–11.85)	0.255
	>70	13 (1.2)	4 (0.4)	1	Ref	1	Ref
Religion	Christianity	464 (43.9)	79 (7.5)	0.34(0.11–1.02)	0.055	2.93(0.98–8.82)	0.124
	Traditional	0 (0.0)	1 (0.1)	**λ**	1.000	**Λ**	1.000
	Islam	383 (36.2)	116 (11.0)	1.65(0.55–4.93)	0.369	2.12(0.64–6.96)	0.218
	Unaffiliated	10 (0.9)	5 (0.5)	1	Ref	1	Ref
Education	No formal education	0 (0.0)	5 (0.5)	**λ**	0.999	**Λ**	0.999
	Primary	2 (0.2)	2 (0.2)	2.99(1.61–5.53)	0.557	1.48(0.75–2.91)	0.154
	Secondary	35 (3.3)	19 (1.8)	1	Ref	1	Ref
	Diploma	169 (16.0)	25 (2.4)	3.67(1.83–7.38)	**0.000** *	1.22(0.55–2.71)	0.619
	Bachelor’s degree	363 (34.3)	66 (6.2)	1.86(1.01–3.42)	**0.001** *	0.83(0.40–1.72)	0.262
	Masters and above	288 (27.2)	84 (7.9)	0.54(0.71–4.17)	**0.046** *	0.20(0.21–1.84)	0.621
Occupation	Health worker	545 (51.5)	50 (4.7)	5.28(3.72–7.48)	**0.000** *	4.83(3.28–7.11)	**0.000** *
	Non-health worker	312 (29.5)	151 (14.3)	1	Ref	1	Ref
Chronic condition	No	787 (74.4)	183 (17.3)	1.11(0.64–1.90)	0.716	1.23(0.67–2.27)	0.502
	Yes	70 (6.6)	18 (1.7)	1	Ref	1	Ref
Prior diagnosis of	No	756 (71.5)	196 (18.5)	0.19(0.08–0.48)	**0.000** *	0.21(0.08–0.54)	**0.001** *
COVID-19	Yes	101 (9.5)	5 (0.5)	1	Ref	1	Ref

cOR = crude odds ratio, aOR = adjusted odds ratio, CI = confidence interval, λ = not included in comparison due to small number

* = statistically significant.

## Discussion

This study aimed to assess the sociodemographic factors influencing the awareness, perception, and willingness of Nigerian adults towards the uptake of COVID-19 vaccine.

Findings from this study showed a positive population-level impact on awareness of COVID-19 vaccination in Nigeria. This is similar to a study carried out in Bangladesh where the majority of respondents were aware of the vaccination activities [5]. Contrary to this, a study carried out in Ethiopia at the commencement of the COVID-19 vaccination roll-out, however showed a low awareness of respondents [22]. The satisfactory score obtained by respondents of this study showed the majority are aware of the COVID-19 vaccination exercise. This may be due to efforts made by the government. Before the commencement of the vaccination exercise in Nigeria, the government, in collaboration with UNICEF and other agencies such as the National Primary Health Care Development Agency (NPHCDA), embarked on raising awareness to address some of the bottlenecks to COVID-19 vaccine uptake. Sensitisation programs and Stakeholder’s engagements with health workers, traditional and community leaders, community youth leaders, market women and those that can influence family and friends were held across the country. There were series of trainings as well as mobilisation and community enlightenment on the safety and importance of getting the COVID-19 vaccine [23]. In this study, being a Muslim or a Christian influences awareness of COVID-19 vaccination more than being unaffiliated. The low awareness of the unaffiliated may also be due to the small size of our sample. Gender and age did not significantly affect the level of awareness in this study, which was similar to a previous study [22]. This may be due to worldwide COVID-19 vaccine advocacy and the indiscriminate intensified efforts of the Nigerian government to ensure all residents, regardless of their demographic status, know about the arrival and commencement of the vaccination exercise. Enhancing situational awareness of information generates knowledge and behaviour that enables people to develop a mental model towards perception, comprehension and prediction of the future status of an event [24].

In this study, findings showed the main sources of COVID-19 vaccination information were the Media (television, radio, and newspapers), health workers and social media platforms. A similar study carried out in Poland showed experts’ materials as a major source of information on COVID-19 vaccines [25]. Health workers and mass media have been identified as important sources of health information for the general population [26, 27]. Doctors and other health workers have been identified as potential communicators through which messages emphasizing the medical and social benefits of the COVID-19 vaccine can be effectively disseminated [28]. To counter misinformation and improve trust, the NPHCDA in partnership with multinational media platforms in Nigeria, has engaged in various initiatives to ensure residents of Nigeria get credible information on COVID-19 vaccination. For instance, there was a range of Facebook frames and Instagram stickers that allowed people to share their support for getting vaccinated with their family and friends. The frames and stickers include banners with a statement such as ’Let’s Get Vaccinated’ or ’I Got My COVID-19 Vaccine’ [29].

Findings from this study also showed varied responses to perception statements, although there was an overall moderately satisfactory response score. Most of the respondents showed a positive perception towards the effectiveness of the vaccine in preventing COVID-19 disease, but there were trust issues as to whether scientists had discovered a safe and effective COVID-19 vaccine or if the Nigerian government had ensured the safety of the vaccine. Almost half of the participants showed fear of the side effects of the vaccine. A survey carried out in France and French-speaking parts of Belgium and Canada showed distrust in the Ministry of Health to ensure the safety of the COVID-19 vaccine [19]. A global cross-sectional study carried out also revealed that half of the participants had safety concerns, especially related to the side effects of the COVID-19 vaccine. In Poland, a study carried out showed varied trust between the vaccines approved in Europe [25]. In a study carried out on trust in the COVID-19 vaccine in the United States, social and societal level factors such as vaccine fast-tracking, government distrust, uncertainty about the content of the vaccine and fear of side effects were highlighted as reasons for distrust of the COVID-19 vaccine [30].

On the influence of sociodemographic characteristics of respondents on perception statements in this study, Nigerian residents between the age range of 40–69 years were more likely to have a positive perception towards COVID-19 vaccine than those below this age range and those above 70 years. This may mean that people within the younger age group think they have better immunity and may not be necessarily vaccinated [31]. The elderly might have a perceived fear of the side effects and likely complications [32, 33]. It may also be because the younger participants are more exposed to vaccine-related misinformation [31]. This may require further investigation. It is worthy of note that trust, confidence, and belief in a program influence the acceptance and successful outcome. The government should intensify efforts for the highest possible outreach through the major sources of information identified in this study. Safety issues with COVID-19 vaccines should be continuously addressed by tailoring clear and reliable targeted messages to promote engagement and acceptance.

The proportion of willingness to get vaccinated in this study was high among the participants and most of them were health workers. Participants with higher education (diploma and above) had higher odds of willingness to get vaccinated compared to participants with secondary school and lower education. This disparity in the willingness to get the COVID-19 vaccination was also found in a study carried out in the United States [34]. Therefore, increasing advocacy and awareness about the COVID-19 vaccine, especially among those with lower educational levels is paramount.

The common predictive factor that influences the awareness, perception, and willingness to get vaccinated in this study were occupation and prior diagnosis for COVID-19. Significantly, respondents who were health workers and those who were previously positive of COVID-19 disease had high awareness, positive perceptions and were more willing to get the vaccine. The influence of prior COVID-19 exposure of respondents to these outcome variables may be heightened due to their real-time experience of COVID-19 disease. Publicized experience sharing by people with a prior diagnosis of COVID-19 disease can give a sound picture of how deadly the disease can be and the essence of getting the vaccine.

Health workers were more likely to be willing to get vaccinated compared to non-health workers. As found in literature, the majority of those unwilling to receive the vaccine were non-health workers [35, 36]. This might imply that the subsequent phases of the COVID-19 vaccination exercise, which involves the public, might experience a low turnout of people. Continuous targeted messages and education focusing on the effectiveness and safety assurance of the vaccine are essential to improve the COVID-19 vaccination exercise and increase uptake. There should be the active involvement of all cadres of health workers (physicians, pharmacists, nurses, community health workers, and others) in ensuring health education of COVID-19 vaccination programs. Healthcare workers play an important role in a successful vaccination program. Their knowledge and awareness determine a recommendation to non-health workers [26].

### Study limitation

Nigerian residents that could not communicate in the English language could not partake in this study. Nigerian residents who do not have android phones could not partake in the study. The convenient sampling method also made respondents skewed towards Southwest of Nigeria than other parts. The electronic sampling method used might have introduced selection bias as those without access to the internet were not involved in this study. These may affect fair representation and findings. However, this is the safest method that could be adopted during this pandemic to reduce the exposure of researchers.

## Conclusion

The current results provide information on the public responses to the COVID-19 vaccination in Nigeria. The study revealed a high level of awareness, willingness to receive the vaccine and moderate perception towards the first phase of vaccination activities. The current study suggests factors such as education, religion, occupation, and prior diagnosis of COVID-19 disease as predictive variables that influence awareness of vaccination exercise. It also indicated occupation and prior diagnosis of the COVID-19 disease as predictive factors that influence the perception and willingness for the vaccine uptake. Health workers were more willing to receive the vaccine than non-health workers. Data from this study identified vaccine distrust and safety concerns as part of perception issues that must be addressed in Nigeria for increased uptake of the COVID-19 vaccine. It is the responsibility of policymakers to ensure continuous COVID-19 vaccine targeted messages are passed through the major channel of information identified in this study (media and health workers).

Findings from this study will enable stakeholders and strategic planners to intensify efforts on related awareness and health education programs that will improve perception and willingness, especially of non-health workers, to ensure a successful vaccination process in subsequent phases.

## Supporting information

S1 File Survey questionnaire. (DOCX)

S2 File Data that supports the finding of this study. (XLSX)

S3 File Geographical distribution of respondents. (DOCX)
