# Supplementary material for: Factors influencing COVID-19 vaccine uptake among adults in Nigeria
Source: PLoS One. 2022 Feb 24;17(2):e0264371. doi: 10.1371/journal.pone.0264371 (PMC8870459; doi:10.1371/journal.pone.0264371)
Supplement: S1 File — (DOCX) [file pone.0264371.s001.docx]

FACTORS INFLUENCING COVID-19 VACCINE UPTAKE AMONG ADULTS IN NIGERIA

*Required

INFORMATION SHEET

Dear Sir/Ma,

You are invited to consider participating in a survey titled “factors influencing covid-19 vaccine uptake among adults in Nigeria.”

With ethics approval from HEALTH RESEARCH COMMITTEE OF LAGO SUNIVERSITY TEACHING HOSPITAL (HREC, LUTH) No: ADM/DCST/HREC/APP/4234. Your participation will provide evidence-based information to help the government plan towards the next phase of the vaccination exercise. This survey is for adults aged 18years and above and participants presently residing in Nigeria.

The duration of your participation if you choose to enroll and remain in the study is expected to be less than 10min. The study is self-sponsored.

INFORMED CONSENT FORM

I have been informed about the study

I understand the purpose and procedures of the study

I have been given an opportunity to answer questions about the study and have had answers to my satisfaction.

I declare that my participation in this study is entirely voluntary and that I may withdraw at anytime without affecting any care that I would usually been titled to.

1. *

*Check all that apply.*

I consent

SOCIO DEMOGRAPHIC CHARACTERISTICS

2. Gender *

*Mark only one oval.*

Female

Male

Prefer not to say

3. Age (kindly state) *

Islam Christianity Traditional Unaffiliated

Other:

5. Highest education level attained *

*Mark only one oval.*

Primary Secondary Diploma Bachelor’s degree

Masters and above

Artisan

6. Occupation *

*Mark only one oval.*

Health worker Government official Lecturer/Researcher Teacher

Legal practitioner

Engineer

Businessman/woman

Unemployed Student Retiree Accountant Others

Lagos State

Ogun State

Oyo State

Other:

8. Kindly state your Local Government Area of residence *

9. Ethnicity *

*Mark only one oval.*

Igbo Hausa Yoruba

Non-Nigerian

Other:

10.

Do you have a chronic condition(s)? *

*Mark only one oval.*

Yes

No

11.

If yes, kindly state the chronic condition(s)

13.

Monthly income *

*Mark only one oval.*

<#18,000

#18,000-#49,000

#50,000-#99,000

#100,000-#300,000

>#300,000

KNOWLEDGE AND AWARENESS ON THE ONGOING COVID 19 VACCINATION

14.

Have you done COVID- 19 test before? *

*Mark only one oval.*

Yes

No

15.

Have you been diagnosed with COVID- 19 before? *

*Mark only one oval.*

Yes

No

16.

If yes, kindly state how long

18.

19.

If yes, kindly state the vaccine(s)

Are you aware of the ongoing first phase COVID 19 vaccination? *

*Mark only one oval.*

Yes

No

20.

If yes, what was your source of information? (tick all that apply) *

*Check all that apply.*

Health worker

Media: Television/Radio/Newspaper

Social media

Friends/family

Other:

21.

Do you think enough awareness has been created about COVID- 19 vaccine in Nigeria? *

*Mark only one oval.*

Yes

No

22.

If No, kindly state how you think awareness can be created

23.

Do you think COVID-19 vaccine is safe? *

*Mark only one oval.*

Yes

No

Notsure

24.

Will you be willing to get COVID 19 vaccine? *

*Mark only one oval.*

Yes

No

25.

If No, kindly state why you are not willing to take the vaccine

PERCEPTION ON COVID 19 VACCINATION

26.

I believe vaccines are effective at preventing diseases *

*Mark only one oval.*

Strongly disagree

Disagree Neutral Agree

Strongly agree

28.

I trust Scientists have developed safe COVID 19 vaccines *

*Mark only one oval.*

Strongly disagree

Disagree Neutral Agree

Strongly agree

29.

I trust Scientists have developed effective COVID 19 vaccines

*Mark only one oval.*

Strongly disagree

Disagree Neutral Agree

Strongly Agree

31.

COVID 19 vaccines developed for Europe and America are safer than the vaccine

developed for Nigeria *

*Mark only one oval.*

Strongly disagree

Disagree Neutral Agree

Strongly agree

32.

COVID 19 vaccines developed for Europe and America are more effective than the

vaccine developed for Nigeria *

*Mark only one oval.*

Strongly disagree

Disagree Neutral Agree

Strongly agree

34.

I don’t believe in the existence of COVID 19 *

*Mark only one oval.*

Strongly disagree

Disagree Neutral Agree

Strongly Agree

35.

I am afraid of likely side effect of COVID 19 vaccine *

*Mark only one oval.*

Strongly disagree

Disagree Neutral Agree

Strongly Agree
