# Supplementary material for: Factors influencing COVID-19 vaccine uptake among adults in Nigeria
Source: PLoS One. 2022 Feb 24;17(2):e0264371. doi: 10.1371/journal.pone.0264371 (PMC8870459; doi:10.1371/journal.pone.0264371)
Supplement: S3 File — (DOCX) [file pone.0264371.s003.docx]

**S4: Geographical distribution of respondents (N=10**5**8)**

|  | | | | |  |
| --- | --- | --- | --- | --- | --- |
| **Region** | | **States** | **Frequency** | **Percent (%)** | |
|  | North Central | Abuja | 20 | 1.9 | |
|  |  | Benue | 2 | 0.2 | |
|  |  | Nasarawa | 4 | 0.4 | |
|  |  | Niger | 1 | 0.1 | |
|  |  | Kwara | 6 | 0.6 | |
|  | North East | Borno | 3 | 0.3 | |
|  |  | Gombe | 1 | 0.1 | |
|  |  | Taraba | 3 | 0.3 | |
|  |  | Yobe | 1 | 0.1 | |
|  | North West | Kaduna | 5 | 0.5 | |
|  |  | Kano | 7 | 0.7 | |
|  |  | Katsina | 2 | 0.2 | |
|  |  | Sokoto | 3 | 0.3 | |
|  | South East | Anambra | 1 | 0.1 | |
|  |  | Ebonyi | 1 | 0.1 | |
|  |  | Enugu | 4 | 0.4 | |
|  | South South | Akwa Ibom State | 1 | 0.1 | |
|  |  | Cross river state | 3 | 0.3 | |
|  |  | Delta State | 2 | 0.2 | |
|  |  | Rivers | 4 | 0.4 | |
|  | South West | Ekiti | 3 | 0.3 | |
|  |  | Lagos State | 810 | 76.6 | |
|  |  | Ogun State | 120 | 11.3 | |
|  |  | Ondo | 1 | 0.1 | |
|  |  | Osun | 6 | 0.6 | |
|  |  | Oyo State | 44 | 4.2 | |
